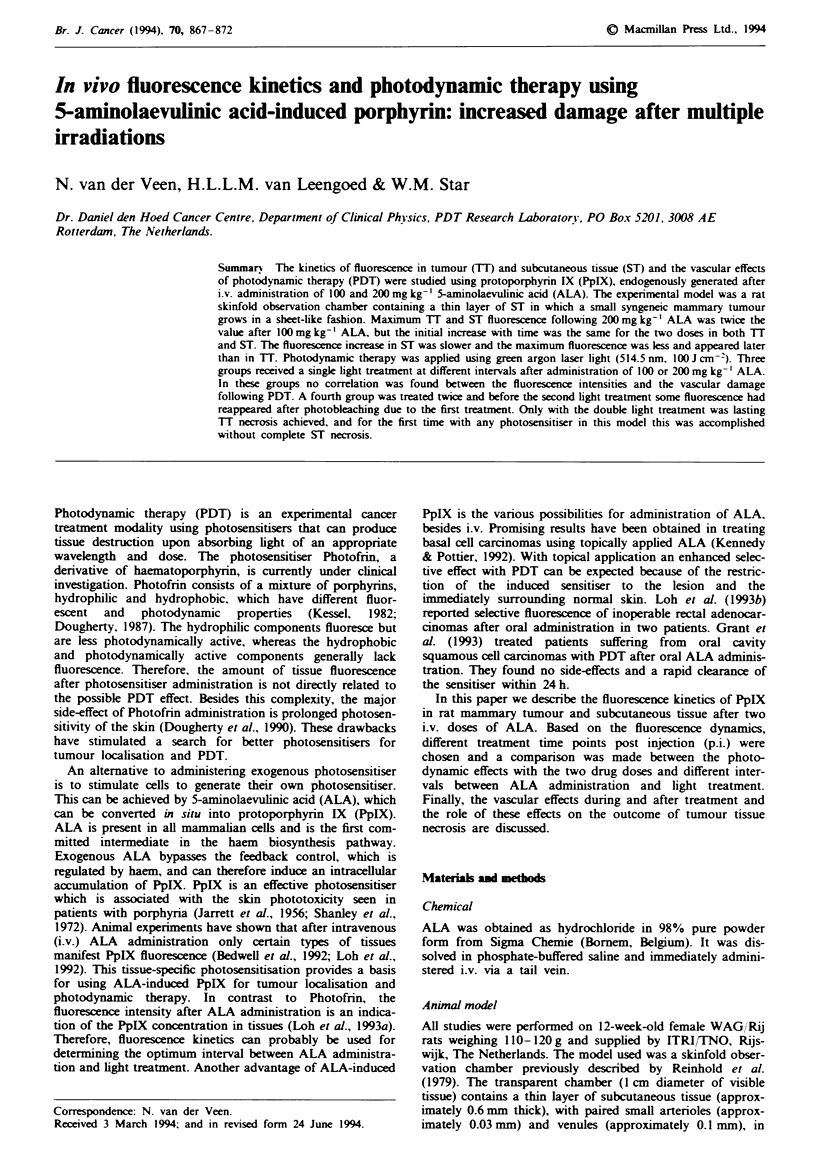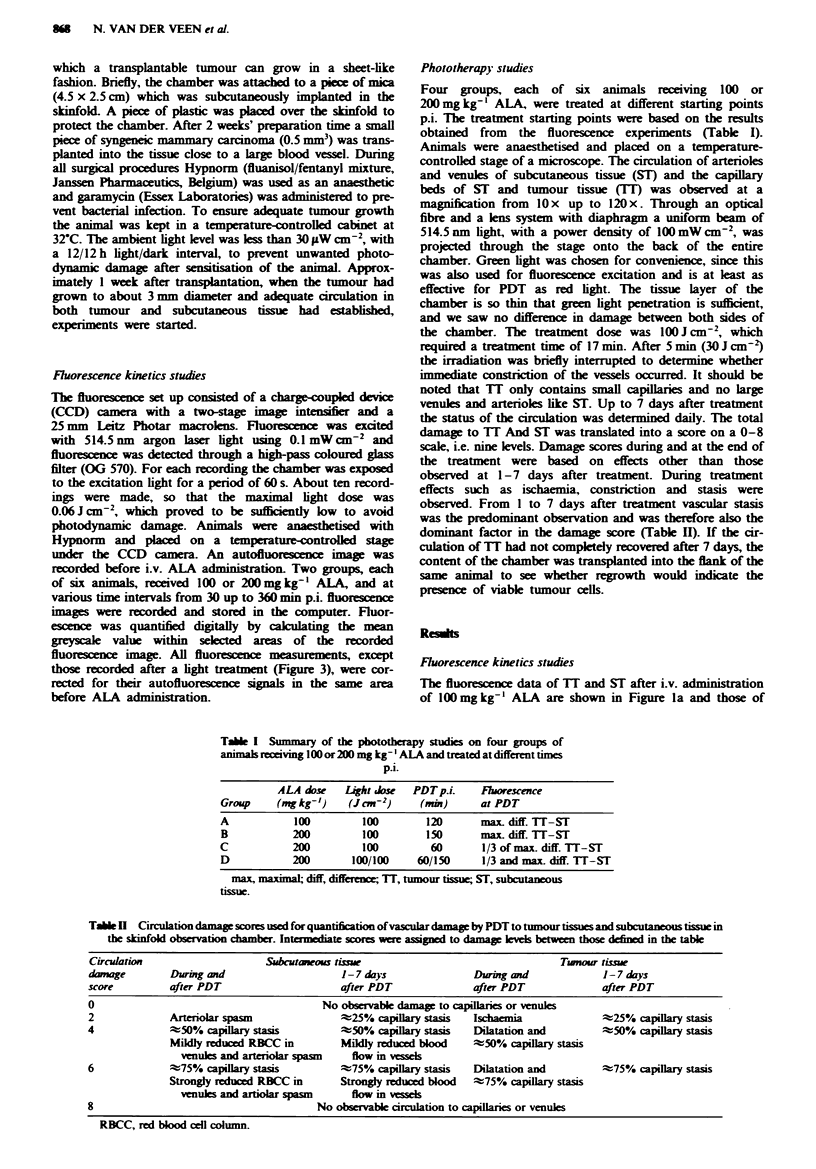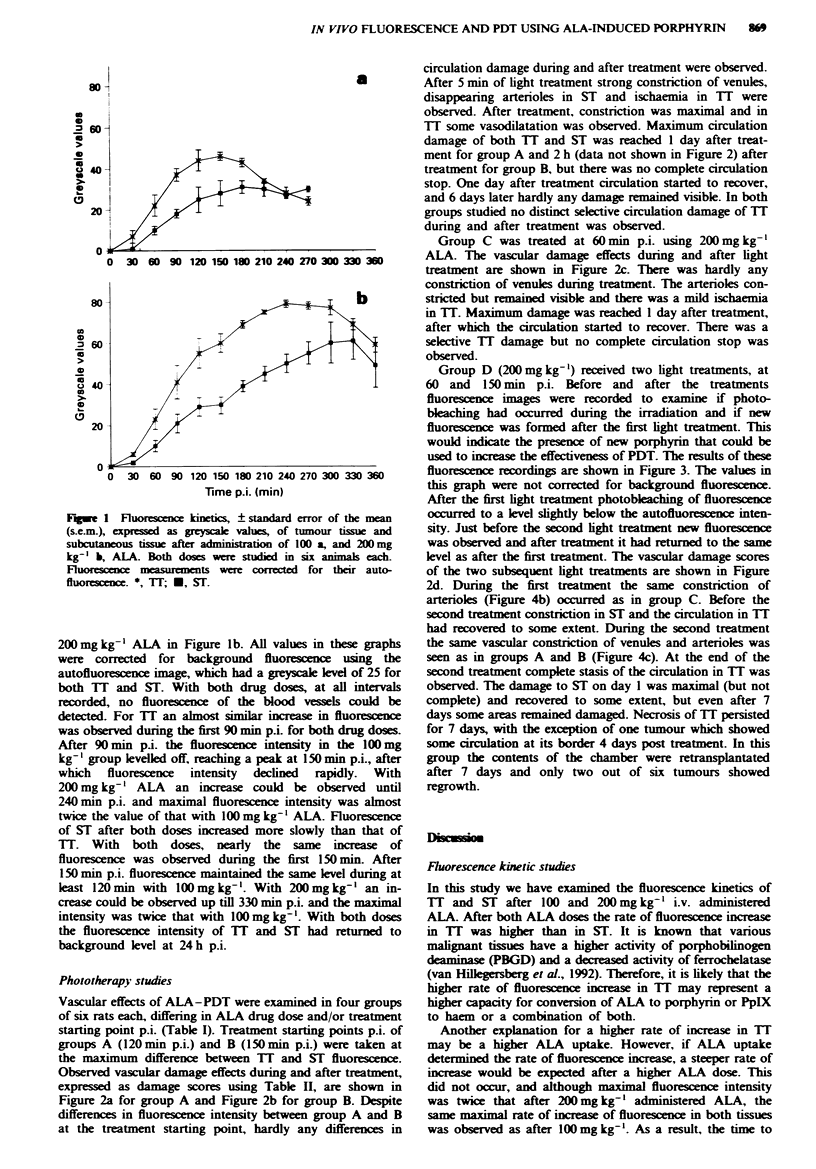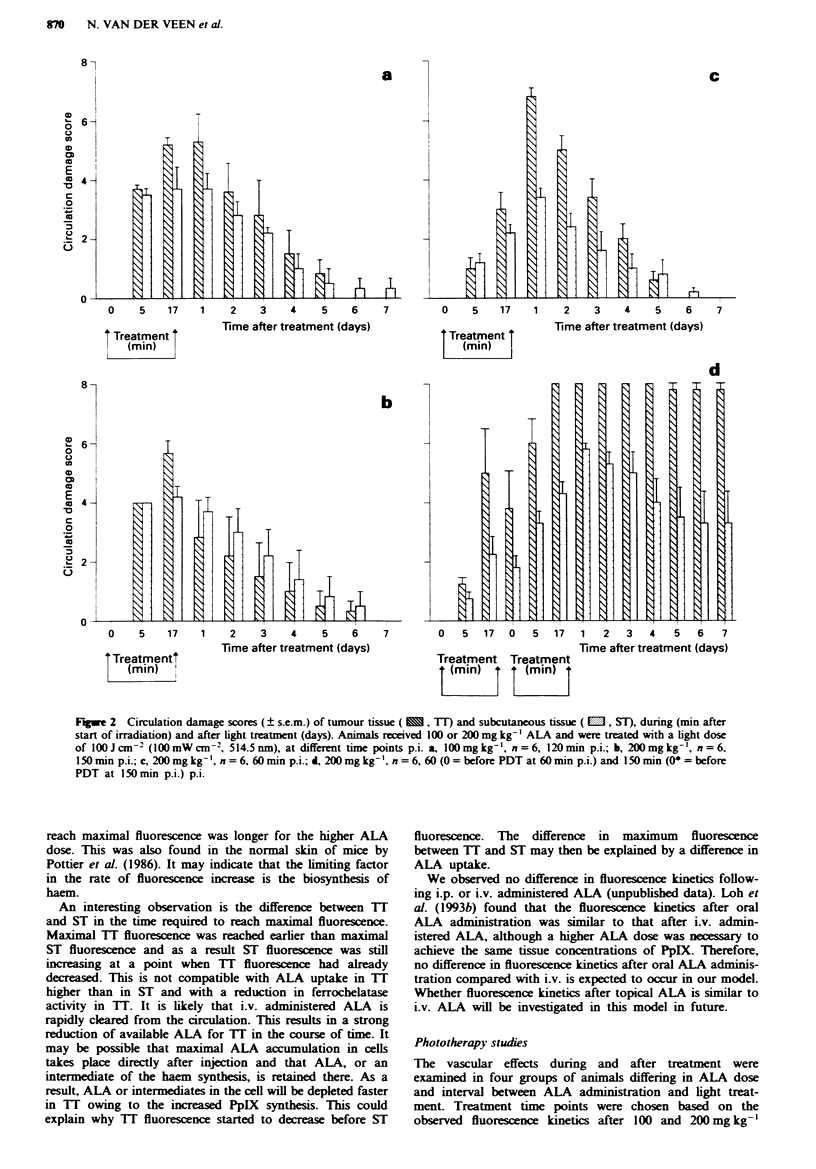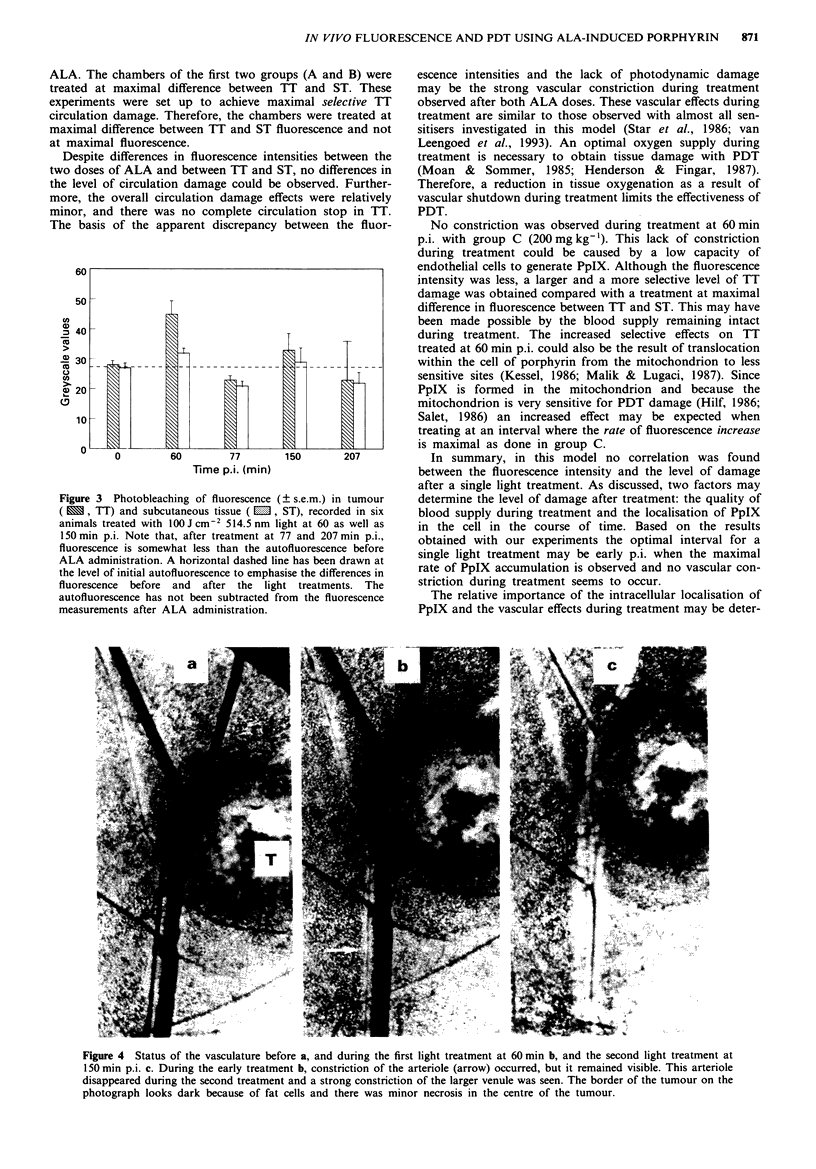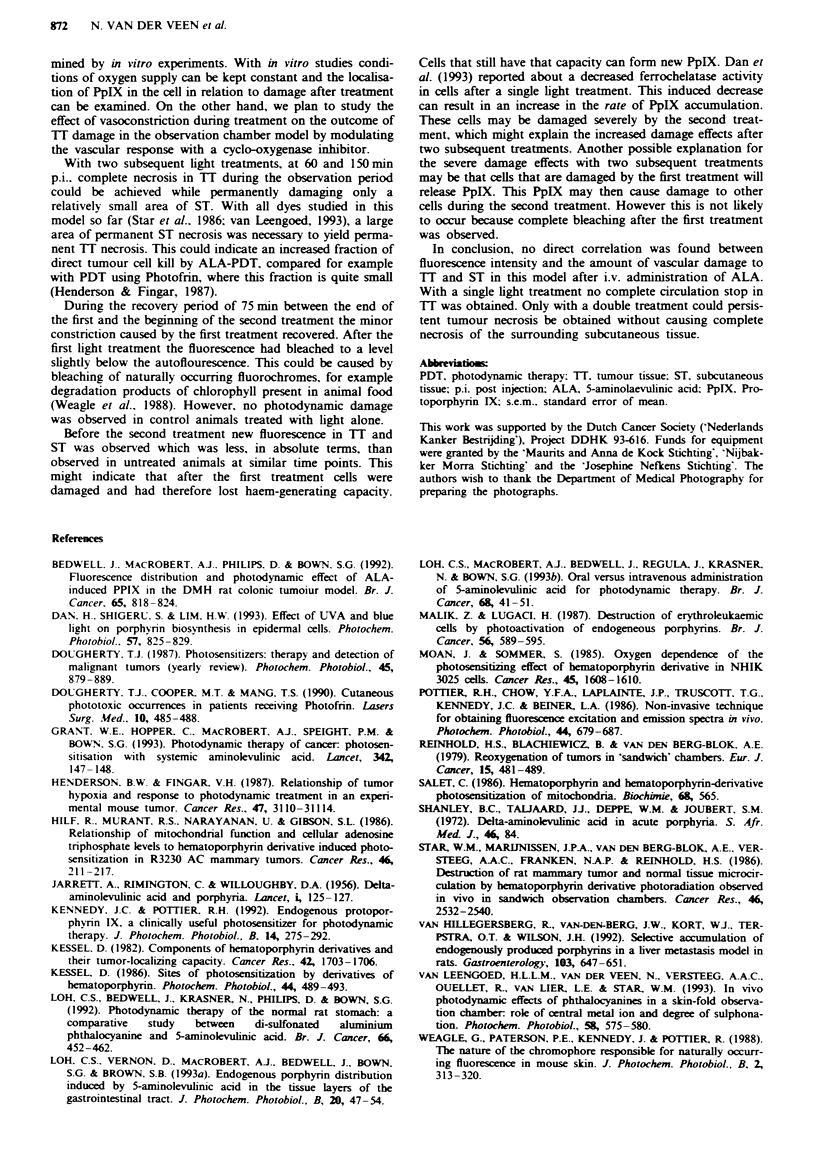# In vivo fluorescence kinetics and photodynamic therapy using 5-aminolaevulinic acid-induced porphyrin: increased damage after multiple irradiations.

**DOI:** 10.1038/bjc.1994.412

**Published:** 1994-11

**Authors:** N. van der Veen, H. L. van Leengoed, W. M. Star

**Affiliations:** Dr. Daniel den Hoed Cancer Centre, Department of Clinical Physics, PDT Research Laboratory, Rotterdam, The Netherlands.

## Abstract

**Images:**


Br. J. Cancer (1994), 70, 867-872 © Macmillan Press Ltd., 1994

### *In vivo* fluorescence kinetics and photodynamic therapy using 5-aminolaevulinic acid-induced porphyrin: increased damage after multiple irradiations

N. van der Veen, H.L.L.M. van Leengoed & W.M. Star

*Dr. Daniel den Hoed Cancer Centre, Department of Clinical Physics, PDT Research Laboratory, PO Box 5201, 3008 AE Rotterdam, The Netherlands.*

**Summary** The kinetics of fluorescence in tumour (TT) and subcutaneous tissue (ST) and the vascular effects of photodynamic therapy (PDT) were studied using protoporphyrin IX (PpIX), endogenously generated after i.v. administration of 100 and 200 mg $kg^{-1}$ 5-aminolaevulinic acid (ALA). The experimental model was a rat skinfold observation chamber containing a thin layer of ST in which a small syngeneic mammary tumour grows in a sheet-like fashion. Maximum TT and ST fluorescence following 200 mg $kg^{-1}$ ALA was twice the value after 100 mg $kg^{-1}$ ALA, but the initial increase with time was the same for the two doses in both TT and ST. The fluorescence increase in ST was slower and the maximum fluorescence was less and appeared later than in TT. Photodynamic therapy was applied using green argon laser light (514.5 nm, 100 J $cm^{-2}$). Three groups received a single light treatment at different intervals after administration of 100 or 200 mg $kg^{-1}$ ALA. In these groups no correlation was found between the fluorescence intensities and the vascular damage following PDT. A fourth group was treated twice and before the second light treatment some fluorescence had reappeared after photobleaching due to the first treatment. Only with the double light treatment was lasting TT necrosis achieved, and for the first time with any photosensitiser in this model this was accomplished without complete ST necrosis.

Photodynamic therapy (PDT) is an experimental cancer treatment modality using photosensitisers that can produce tissue destruction upon absorbing light of an appropriate wavelength and dose. The photosensitiser Photofrin, a derivative of haematoporphyrin, is currently under clinical investigation. Photofrin consists of a mixture of porphyrins, hydrophilic and hydrophobic, which have different fluorescent and photodynamic properties (Kessel, 1982; Dougherty, 1987). The hydrophilic components fluoresce but are less photodynamically active, whereas the hydrophobic and photodynamically active components generally lack fluorescence. Therefore, the amount of tissue fluorescence after photosensitiser administration is not directly related to the possible PDT effect. Besides this complexity, the major side-effect of Photofrin administration is prolonged photosensitivity of the skin (Dougherty *et al.*, 1990). These drawbacks have stimulated a search for better photosensitisers for tumour localisation and PDT.

An alternative to administering exogenous photosensitiser is to stimulate cells to generate their own photosensitiser. This can be achieved by 5-aminolaevulinic acid (ALA), which can be converted *in situ* into protoporphyrin IX (PpIX). ALA is present in all mammalian cells and is the first committed intermediate in the haem biosynthesis pathway. Exogenous ALA bypasses the feedback control, which is regulated by haem, and can therefore induce an intracellular accumulation of PpIX. PpIX is an effective photosensitiser which is associated with the skin phototoxicity seen in patients with porphyria (Jarrett *et al.*, 1956; Shanley *et al.*, 1972). Animal experiments have shown that after intravenous (i.v.) ALA administration only certain types of tissues manifest PpIX fluorescence (Bedwell *et al.*, 1992; Loh *et al.*, 1992). This tissue-specific photosensitisation provides a basis for using ALA-induced PpIX for tumour localisation and photodynamic therapy. In contrast to Photofrin, the fluorescence intensity after ALA administration is an indication of the PpIX concentration in tissues (Loh *et al.*, 1993*a*). Therefore, fluorescence kinetics can probably be used for determining the optimum interval between ALA administration and light treatment. Another advantage of ALA-induced PpIX is the various possibilities for administration of ALA, besides i.v. Promising results have been obtained in treating basal cell carcinomas using topically applied ALA (Kennedy & Pottier, 1992). With topical application an enhanced selective effect with PDT can be expected because of the restriction of the induced sensitiser to the lesion and the immediately surrounding normal skin. Loh *et al.* (1993*b*) reported selective fluorescence of inoperable rectal adenocarcinomas after oral administration in two patients. Grant *et al.* (1993) treated patients suffering from oral cavity squamous cell carcinomas with PDT after oral ALA administration. They found no side-effects and a rapid clearance of the sensitiser within 24 h.

In this paper we describe the fluorescence kinetics of PpIX in rat mammary tumour and subcutaneous tissue after two i.v. doses of ALA. Based on the fluorescence dynamics, different treatment time points post injection (p.i.) were chosen and a comparison was made between the photodynamic effects with the two drug doses and different intervals between ALA administration and light treatment. Finally, the vascular effects during and after treatment and the role of these effects on the outcome of tumour tissue necrosis are discussed.

### Materials and methods

### *Chemical*

ALA was obtained as hydrochloride in 98% pure powder form from Sigma Chemie (Bornem, Belgium). It was dissolved in phosphate-buffered saline and immediately administered i.v. via a tail vein.

### *Animal model*

All studies were performed on 12-week-old female WAG/Rij rats weighing 110-120 g and supplied by ITRI/TNO, Rijswijk, The Netherlands. The model used was a skinfold observation chamber previously described by Reinhold *et al.* (1979). The transparent chamber (1 cm diameter of visible tissue) contains a thin layer of subcutaneous tissue (approximately 0.6 mm thick), with paired small arterioles (approximately 0.03 mm) and venules (approximately 0.1 mm), in

Correspondence: N. van der Veen.
Received 3 March 1994; and in revised form 24 June 1994.

which a transplantable tumour can grow in a sheet-like fashion. Briefly, the chamber was attached to a piece of mica (4.5 x 2.5 cm) which was subcutaneously implanted in the skinfold. A piece of plastic was placed over the skinfold to protect the chamber. After 2 weeks' preparation time a small piece of syngeneic mammary carcinoma (0.5 $mm^3$) was transplanted into the tissue close to a large blood vessel. During all surgical procedures Hypnorm (fluanisol/fentanyl mixture, Janssen Pharmaceutics, Belgium) was used as an anaesthetic and garamycin (Essex Laboratories) was administered to prevent bacterial infection. To ensure adequate tumour growth the animal was kept in a temperature-controlled cabinet at 32°C. The ambient light level was less than 30 $\mu W\ cm^{-2}$, with a 12/12 h light/dark interval, to prevent unwanted photodynamic damage after sensitisation of the animal. Approximately 1 week after transplantation, when the tumour had grown to about 3 mm diameter and adequate circulation in both tumour and subcutaneous tissue had established, experiments were started.

### Fluorescence kinetics studies

The fluorescence set up consisted of a charge-coupled device (CCD) camera with a two-stage image intensifier and a 25 mm Leitz Photar macrolens. Fluorescence was excited with 514.5 nm argon laser light using 0.1 $mW\ cm^{-2}$ and fluorescence was detected through a high-pass coloured glass filter (OG 570). For each recording the chamber was exposed to the excitation light for a period of 60 s. About ten recordings were made, so that the maximal light dose was 0.06 $J\ cm^{-2}$, which proved to be sufficiently low to avoid photodynamic damage. Animals were anaesthetised with Hypnorm and placed on a temperature-controlled stage under the CCD camera. An autofluorescence image was recorded before i.v. ALA administration. Two groups, each of six animals, received 100 or 200 $mg\ kg^{-1}$ ALA, and at various time intervals from 30 up to 360 min p.i. fluorescence images were recorded and stored in the computer. Fluorescence was quantified digitally by calculating the mean greyscale value within selected areas of the recorded fluorescence image. All fluorescence measurements, except those recorded after a light treatment (Figure 3), were corrected for their autofluorescence signals in the same area before ALA administration.

### Phototherapy studies

Four groups, each of six animals receiving 100 or 200 $mg\ kg^{-1}$ ALA, were treated at different starting points p.i. The treatment starting points were based on the results obtained from the fluorescence experiments (Table I). Animals were anaesthetised and placed on a temperature-controlled stage of a microscope. The circulation of arterioles and venules of subcutaneous tissue (ST) and the capillary beds of ST and tumour tissue (TT) was observed at a magnification from 10× up to 120×. Through an optical fibre and a lens system with diaphragm a uniform beam of 514.5 nm light, with a power density of 100 $mW\ cm^{-2}$, was projected through the stage onto the back of the entire chamber. Green light was chosen for convenience, since this was also used for fluorescence excitation and is at least as effective for PDT as red light. The tissue layer of the chamber is so thin that green light penetration is sufficient, and we saw no difference in damage between both sides of the chamber. The treatment dose was 100 $J\ cm^{-2}$, which required a treatment time of 17 min. After 5 min (30 $J\ cm^{-2}$) the irradiation was briefly interrupted to determine whether immediate constriction of the vessels occurred. It should be noted that TT only contains small capillaries and no large venules and arterioles like ST. Up to 7 days after treatment the status of the circulation was determined daily. The total damage to TT And ST was translated into a score on a 0-8 scale, i.e. nine levels. Damage scores during and at the end of the treatment were based on effects other than those observed at 1-7 days after treatment. During treatment effects such as ischaemia, constriction and stasis were observed. From 1 to 7 days after treatment vascular stasis was the predominant observation and was therefore also the dominant factor in the damage score (Table II). If the circulation of TT had not completely recovered after 7 days, the content of the chamber was transplanted into the flank of the same animal to see whether regrowth would indicate the presence of viable tumour cells.

### Results

### Fluorescence kinetics studies

The fluorescence data of TT and ST after i.v. administration of 100 $mg\ kg^{-1}$ ALA are shown in Figure 1a and those of

**Table I** Summary of the phototherapy studies on four groups of animals receiving 100 or 200 $mg\ kg^{-1}$ ALA and treated at different times p.i.

| *Group* | *ALA dose ($mg\ kg^{-1}$)* | *Light dose ($J\ cm^{-2}$)* | *PDT p.i. (min)* | *Fluorescence at PDT* |
|---|---|---|---|---|
| A | 100 | 100 | 120 | max. diff. TT-ST |
| B | 200 | 100 | 150 | max. diff. TT-ST |
| C | 200 | 100 | 60 | 1/3 of max. diff. TT-ST |
| D | 200 | 100/100 | 60/150 | 1/3 and max. diff. TT-ST |

max, maximal; diff, difference; TT, tumour tissue; ST, subcutaneous tissue.

**Table II** Circulation damage scores used for quantification of vascular damage by PDT to tumour tissues and subcutaneous tissue in the skinfold observation chamber. Intermediate scores were assigned to damage levels between those defined in the table

| *Circulation damage score* | *Subcutaneous tissue: During and after PDT* | *Subcutaneous tissue: 1-7 days after PDT* | *Tumour tissue: During and after PDT* | *Tumour tissue: 1-7 days after PDT* |
|---|---|---|---|---|
| 0 | No observable damage to capillaries or venules | | | |
| 2 | Arteriolar spasm | ≈25% capillary stasis | Ischaemia | ≈25% capillary stasis |
| 4 | ≈50% capillary stasis<br>Mildly reduced RBCC in venules and arteriolar spasm | ≈50% capillary stasis<br>Mildly reduced blood flow in vessels | Dilatation and ≈50% capillary stasis | ≈50% capillary stasis |
| 6 | ≈75% capillary stasis<br>Strongly reduced RBCC in venules and artiolar spasm | ≈75% capillary stasis<br>Strongly reduced blood flow in vessels | Dilatation and ≈75% capillary stasis | ≈75% capillary stasis |
| 8 | No observable circulation to capillaries or venules | | | |

RBCC, red blood cell column.

**Figure 1** Fluorescence kinetics, ± standard error of the mean (s.e.m.), expressed as greyscale values, of tumour tissue and subcutaneous tissue after administration of 100 **a**, and 200 mg $kg^{-1}$ **b**, ALA. Both doses were studied in six animals each. Fluorescence measurements were corrected for their autofluorescence. *, TT; ■, ST.

200 mg $kg^{-1}$ ALA in Figure 1b. All values in these graphs were corrected for background fluorescence using the autofluorescence image, which had a greyscale level of 25 for both TT and ST. With both drug doses, at all intervals recorded, no fluorescence of the blood vessels could be detected. For TT an almost similar increase in fluorescence was observed during the first 90 min p.i. for both drug doses. After 90 min p.i. the fluorescence intensity in the 100 mg $kg^{-1}$ group levelled off, reaching a peak at 150 min p.i., after which fluorescence intensity declined rapidly. With 200 mg $kg^{-1}$ ALA an increase could be observed until 240 min p.i. and maximal fluorescence intensity was almost twice the value of that with 100 mg $kg^{-1}$ ALA. Fluorescence of ST after both doses increased more slowly than that of TT. With both doses, nearly the same increase of fluorescence was observed during the first 150 min. After 150 min p.i. fluorescence maintained the same level during at least 120 min with 100 mg $kg^{-1}$. With 200 mg $kg^{-1}$ an increase could be observed up till 330 min p.i. and the maximal intensity was twice that with 100 mg $kg^{-1}$. With both doses the fluorescence intensity of TT and ST had returned to background level at 24 h p.i.

*Phototherapy studies*

Vascular effects of ALA-PDT were examined in four groups of six rats each, differing in ALA drug dose and/or treatment starting point p.i. (Table I). Treatment starting points p.i. of groups A (120 min p.i.) and B (150 min p.i.) were taken at the maximum difference between TT and ST fluorescence. Observed vascular damage effects during and after treatment, expressed as damage scores using Table II, are shown in Figure 2a for group A and Figure 2b for group B. Despite differences in fluorescence intensity between group A and B at the treatment starting point, hardly any differences in circulation damage during and after treatment were observed. After 5 min of light treatment strong constriction of venules, disappearing arterioles in ST and ischaemia in TT were observed. After treatment, constriction was maximal and in TT some vasodilatation was observed. Maximum circulation damage of both TT and ST was reached 1 day after treatment for group A and 2 h (data not shown in Figure 2) after treatment for group B, but there was no complete circulation stop. One day after treatment circulation started to recover, and 6 days later hardly any damage remained visible. In both groups studied no distinct selective circulation damage of TT during and after treatment was observed.

Group C was treated at 60 min p.i. using 200 mg $kg^{-1}$ ALA. The vascular damage effects during and after light treatment are shown in Figure 2c. There was hardly any constriction of venules during treatment. The arterioles constricted but remained visible and there was a mild ischaemia in TT. Maximum damage was reached 1 day after treatment, after which the circulation started to recover. There was a selective TT damage but no complete circulation stop was observed.

Group D (200 mg $kg^{-1}$) received two light treatments, at 60 and 150 min p.i. Before and after the treatments fluorescence images were recorded to examine if photobleaching had occurred during the irradiation and if new fluorescence was formed after the first light treatment. This would indicate the presence of new porphyrin that could be used to increase the effectiveness of PDT. The results of these fluorescence recordings are shown in Figure 3. The values in this graph were not corrected for background fluorescence. After the first light treatment photobleaching of fluorescence occurred to a level slightly below the autofluorescence intensity. Just before the second light treatment new fluorescence was observed and after treatment it had returned to the same level as after the first treatment. The vascular damage scores of the two subsequent light treatments are shown in Figure 2d. During the first treatment the same constriction of arterioles (Figure 4b) occurred as in group C. Before the second treatment constriction in ST and the circulation in TT had recovered to some extent. During the second treatment the same vascular constriction of venules and arterioles was seen as in groups A and B (Figure 4c). At the end of the second treatment complete stasis of the circulation in TT was observed. The damage to ST on day 1 was maximal (but not complete) and recovered to some extent, but even after 7 days some areas remained damaged. Necrosis of TT persisted for 7 days, with the exception of one tumour which showed some circulation at its border 4 days post treatment. In this group the contents of the chamber were retransplantated after 7 days and only two out of six tumours showed regrowth.

### Discussion

*Fluorescence kinetic studies*

In this study we have examined the fluorescence kinetics of TT and ST after 100 and 200 mg $kg^{-1}$ i.v. administered ALA. After both ALA doses the rate of fluorescence increase in TT was higher than in ST. It is known that various malignant tissues have a higher activity of porphobilinogen deaminase (PBGD) and a decreased activity of ferrochelatase (van Hillegersberg *et al.*, 1992). Therefore, it is likely that the higher rate of fluorescence increase in TT may represent a higher capacity for conversion of ALA to porphyrin or PpIX to haem or a combination of both.

Another explanation for a higher rate of increase in TT may be a higher ALA uptake. However, if ALA uptake determined the rate of fluorescence increase, a steeper rate of increase would be expected after a higher ALA dose. This did not occur, and although maximal fluorescence intensity was twice that after 200 mg $kg^{-1}$ administered ALA, the same maximal rate of increase of fluorescence in both tissues was observed as after 100 mg $kg^{-1}$. As a result, the time to

**Figure 2** Circulation damage scores (± s.e.m.) of tumour tissue ( , TT) and subcutaneous tissue ( , ST), during (min after start of irradiation) and after light treatment (days). Animals received 100 or 200 mg kg$^{-1}$ ALA and were treated with a light dose of 100 J cm$^{-2}$ (100 mW cm$^{-2}$, 514.5 nm), at different time points p.i. **a**, 100 mg kg$^{-1}$, $n = 6$, 120 min p.i.; **b**, 200 mg kg$^{-1}$, $n = 6$, 150 min p.i.; **c**, 200 mg kg$^{-1}$, $n = 6$, 60 min p.i.; **d**, 200 mg kg$^{-1}$, $n = 6$, 60 (0 = before PDT at 60 min p.i.) and 150 min (0* = before PDT at 150 min p.i.) p.i.

reach maximal fluorescence was longer for the higher ALA dose. This was also found in the normal skin of mice by Pottier *et al.* (1986). It may indicate that the limiting factor in the rate of fluorescence increase is the biosynthesis of haem.

An interesting observation is the difference between TT and ST in the time required to reach maximal fluorescence. Maximal TT fluorescence was reached earlier than maximal ST fluorescence and as a result ST fluorescence was still increasing at a point when TT fluorescence had already decreased. This is not compatible with ALA uptake in TT higher than in ST and with a reduction in ferrochelatase activity in TT. It is likely that i.v. administered ALA is rapidly cleared from the circulation. This results in a strong reduction of available ALA for TT in the course of time. It may be possible that maximal ALA accumulation in cells takes place directly after injection and that ALA, or an intermediate of the haem synthesis, is retained there. As a result, ALA or intermediates in the cell will be depleted faster in TT owing to the increased PpIX synthesis. This could explain why TT fluorescence started to decrease before ST fluorescence. The difference in maximum fluorescence between TT and ST may then be explained by a difference in ALA uptake.

We observed no difference in fluorescence kinetics following i.p. or i.v. administered ALA (unpublished data). Loh *et al.* (1993*b*) found that the fluorescence kinetics after oral ALA administration was similar to that after i.v. administered ALA, although a higher ALA dose was necessary to achieve the same tissue concentrations of PpIX. Therefore, no difference in fluorescence kinetics after oral ALA administration compared with i.v. is expected to occur in our model. Whether fluorescence kinetics after topical ALA is similar to i.v. ALA will be investigated in this model in future.

### *Phototherapy studies*

The vascular effects during and after treatment were examined in four groups of animals differing in ALA dose and interval between ALA administration and light treatment. Treatment time points were chosen based on the observed fluorescence kinetics after 100 and 200 mg kg$^{-1}$

ALA. The chambers of the first two groups (A and B) were treated at maximal difference between TT and ST. These experiments were set up to achieve maximal *selective* TT circulation damage. Therefore, the chambers were treated at maximal difference between TT and ST fluorescence and not at maximal fluorescence.

Despite differences in fluorescence intensities between the two doses of ALA and between TT and ST, no differences in the level of circulation damage could be observed. Furthermore, the overall circulation damage effects were relatively minor, and there was no complete circulation stop in TT. The basis of the apparent discrepancy between the fluorescence intensities and the lack of photodynamic damage may be the strong vascular constriction during treatment observed after both ALA doses. These vascular effects during treatment are similar to those observed with almost all sensitisers investigated in this model (Star *et al.*, 1986; van Leengoed *et al.*, 1993). An optimal oxygen supply during treatment is necessary to obtain tissue damage with PDT (Moan & Sommer, 1985; Henderson & Fingar, 1987). Therefore, a reduction in tissue oxygenation as a result of vascular shutdown during treatment limits the effectiveness of PDT.

**Figure 3** Photobleaching of fluorescence (± s.e.m.) in tumour ( , TT) and subcutaneous tissue ( , ST), recorded in six animals treated with 100 J $cm^{-2}$ 514.5 nm light at 60 as well as 150 min p.i. Note that, after treatment at 77 and 207 min p.i., fluorescence is somewhat less than the autofluorescence before ALA administration. A horizontal dashed line has been drawn at the level of initial autofluorescence to emphasise the differences in fluorescence before and after the light treatments. The autofluorescence has not been subtracted from the fluorescence measurements after ALA administration.

No constriction was observed during treatment at 60 min p.i. with group C (200 mg $kg^{-1}$). This lack of constriction during treatment could be caused by a low capacity of endothelial cells to generate PpIX. Although the fluorescence intensity was less, a larger and a more selective level of TT damage was obtained compared with a treatment at maximal difference in fluorescence between TT and ST. This may have been made possible by the blood supply remaining intact during treatment. The increased selective effects on TT treated at 60 min p.i. could also be the result of translocation within the cell of porphyrin from the mitochondrion to less sensitive sites (Kessel, 1986; Malik & Lugaci, 1987). Since PpIX is formed in the mitochondrion and because the mitochondrion is very sensitive for PDT damage (Hilf, 1986; Salet, 1986) an increased effect may be expected when treating at an interval where the *rate* of fluorescence *increase* is maximal as done in group C.

In summary, in this model no correlation was found between the fluorescence intensity and the level of damage after a single light treatment. As discussed, two factors may determine the level of damage after treatment: the quality of blood supply during treatment and the localisation of PpIX in the cell in the course of time. Based on the results obtained with our experiments the optimal interval for a single light treatment may be early p.i. when the maximal rate of PpIX accumulation is observed and no vascular constriction during treatment seems to occur.

The relative importance of the intracellular localisation of PpIX and the vascular effects during treatment may be deter-

**Figure 4** Status of the vasculature before **a**, and during the first light treatment at 60 min **b**, and the second light treatment at 150 min p.i. **c**. During the early treatment **b**, constriction of the arteriole (arrow) occurred, but it remained visible. This arteriole disappeared during the second treatment and a strong constriction of the larger venule was seen. The border of the tumour on the photograph looks dark because of fat cells and there was minor necrosis in the centre of the tumour.

mined by *in vitro* experiments. With *in vitro* studies conditions of oxygen supply can be kept constant and the localisation of PpIX in the cell in relation to damage after treatment can be examined. On the other hand, we plan to study the effect of vasoconstriction during treatment on the outcome of TT damage in the observation chamber model by modulating the vascular response with a cyclo-oxygenase inhibitor.

With two subsequent light treatments, at 60 and 150 min p.i., complete necrosis in TT during the observation period could be achieved while permanently damaging only a relatively small area of ST. With all dyes studied in this model so far (Star *et al.*, 1986; van Leengoed, 1993), a large area of permanent ST necrosis was necessary to yield permanent TT necrosis. This could indicate an increased fraction of direct tumour cell kill by ALA-PDT, compared for example with PDT using Photofrin, where this fraction is quite small (Henderson & Fingar, 1987).

During the recovery period of 75 min between the end of the first and the beginning of the second treatment the minor constriction caused by the first treatment recovered. After the first light treatment the fluorescence had bleached to a level slightly below the autoflourescence. This could be caused by bleaching of naturally occurring fluorochromes, for example degradation products of chlorophyll present in animal food (Weagle *et al.*, 1988). However, no photodynamic damage was observed in control animals treated with light alone.

Before the second treatment new fluorescence in TT and ST was observed which was less, in absolute terms, than observed in untreated animals at similar time points. This might indicate that after the first treatment cells were damaged and had therefore lost haem-generating capacity. Cells that still have that capacity can form new PpIX. Dan *et al.* (1993) reported about a decreased ferrochelatase activity in cells after a single light treatment. This induced decrease can result in an increase in the *rate* of PpIX accumulation. These cells may be damaged severely by the second treatment, which might explain the increased damage effects after two subsequent treatments. Another possible explanation for the severe damage effects with two subsequent treatments may be that cells that are damaged by the first treatment will release PpIX. This PpIX may then cause damage to other cells during the second treatment. However this is not likely to occur because complete bleaching after the first treatment was observed.

In conclusion, no direct correlation was found between fluorescence intensity and the amount of vascular damage to TT and ST in this model after i.v. administration of ALA. With a single light treatment no complete circulation stop in TT was obtained. Only with a double treatment could persistent tumour necrosis be obtained without causing complete necrosis of the surrounding subcutaneous tissue.

**Abbreviations:**
PDT, photodynamic therapy; TT, tumour tissue; ST, subcutaneous tissue; p.i. post injection; ALA, 5-aminolaevulinic acid; PpIX, Protoporphyrin IX; s.e.m., standard error of mean.

This work was supported by the Dutch Cancer Society ('Nederlands Kanker Bestrijding'), Project DDHK 93-616. Funds for equipment were granted by the 'Maurits and Anna de Kock Stichting', 'Nijbakker Morra Stichting' and the 'Josephine Nefkens Stichting'. The authors wish to thank the Department of Medical Photography for preparing the photographs.

### References

BEDWELL, J., MACROBERT, A.J., PHILIPS, D. & BOWN, S.G. (1992). Fluorescence distribution and photodynamic effect of ALA-induced PPIX in the DMH rat colonic tumoiur model. *Br. J. Cancer*, **65**, 818-824.

DAN, H., SHIGERU, S. & LIM, H.W. (1993). Effect of UVA and blue light on porphyrin biosynthesis in epidermal cells. *Photochem. Photobiol.*, **57**, 825-829.

DOUGHERTY, T.J. (1987). Photosensitizers: therapy and detection of malignant tumors (yearly review). *Photochem. Photobiol.*, **45**, 879-889.

DOUGHERTY, T.J., COOPER, M.T. & MANG, T.S. (1990). Cutaneous phototoxic occurrences in patients receiving Photofrin. *Lasers Surg. Med.*, **10**, 485-488.

GRANT, W.E., HOPPER, C., MACROBERT, A.J., SPEIGHT, P.M. & BOWN, S.G. (1993). Photodynamic therapy of cancer: photosensitisation with systemic aminolevulinic acid. *Lancet*, **342**, 147-148.

HENDERSON, B.W. & FINGAR, V.H. (1987). Relationship of tumor hypoxia and response to photodynamic treatment in an experimental mouse tumor. *Cancer Res.*, **47**, 3110-31114.

HILF, R., MURANT, R.S., NARAYANAN, U. & GIBSON, S.L. (1986). Relationship of mitochondrial function and cellular adenosine triphosphate levels to hematoporphyrin derivative induced photosensitization in R3230 AC mammary tumors. *Cancer Res.*, **46**, 211-217.

JARRETT, A., RIMINGTON, C. & WILLOUGHBY, D.A. (1956). Delta-aminolevulinic acid and porphyria. *Lancet*, **i**, 125-127.

KENNEDY, J.C. & POTTIER, R.H. (1992). Endogenous protoporphyrin IX, a clinically useful photosensitizer for photodynamic therapy. *J. Photochem. Photobiol., B*, **14**, 275-292.

KESSEL, D. (1982). Components of hematoporphyrin derivatives and their tumor-localizing capacity. *Cancer Res.*, **42**, 1703-1706.

KESSEL, D. (1986). Sites of photosensitization by derivatives of hematoporphyrin. *Photochem. Photobiol.*, **44**, 489-493.

LOH, C.S., BEDWELL, J., KRASNER, N., PHILIPS, D. & BOWN, S.G. (1992). Photodynamic therapy of the normal rat stomach: a comparative study between di-sulfonated aluminium phthalocyanine and 5-aminolevulinic acid. *Br. J. Cancer*, **66**, 452-462.

LOH, C.S., VERNON, D., MACROBERT, A.J., BEDWELL, J., BOWN, S.G. & BROWN, S.B. (1993*a*). Endogenous porphyrin distribution induced by 5-aminolevulinic acid in the tissue layers of the gastrointestinal tract. *J. Photochem. Photobiol., B*, **20**, 47-54.

LOH, C.S., MACROBERT, A.J., BEDWELL, J., REGULA, J., KRASNER, N. & BOWN, S.G. (1993*b*). Oral versus intravenous administration of 5-aminolevulinic acid for photodynamic therapy. *Br. J. Cancer*, **68**, 41-51.

MALIK, Z. & LUGACI, H. (1987). Destruction of erythroleukaemic cells by photoactivation of endogeneous porphyrins. *Br. J. Cancer*, **56**, 589-595.

MOAN, J. & SOMMER, S. (1985). Oxygen dependence of the photosensitizing effect of hematoporphyrin derivative in NHIK 3025 cells. *Cancer Res.*, **45**, 1608-1610.

POTTIER, R.H., CHOW, Y.F.A., LAPLAINTE, J.P., TRUSCOTT, T.G., KENNEDY, J.C. & BEINER, L.A. (1986). Non-invasive technique for obtaining fluorescence excitation and emission spectra *in vivo*. *Photochem. Photobiol.*, **44**, 679-687.

REINHOLD, H.S., BLACHIEWICZ, B. & VAN DEN BERG-BLOK, A.E. (1979). Reoxygenation of tumors in 'sandwich' chambers. *Eur. J. Cancer*, **15**, 481-489.

SALET, C. (1986). Hematoporphyrin and hematoporphyrin-derivative photosensitization of mitochondria. *Biochimie*, **68**, 565.

SHANLEY, B.C., TALJAARD, J.J., DEPPE, W.M. & JOUBERT, S.M. (1972). Delta-aminolevulinic acid in acute porphyria. *S. Afr. Med. J.*, **46**, 84.

STAR, W.M., MARIJNISSEN, J.P.A., VAN DEN BERG-BLOK, A.E., VERSTEEG, A.A.C., FRANKEN, N.A.P. & REINHOLD, H.S. (1986). Destruction of rat mammary tumor and normal tissue microcirculation by hematoporphyrin derivative photoradiation observed in vivo in sandwich observation chambers. *Cancer Res.*, **46**, 2532-2540.

VAN HILLEGERSBERG, R., VAN-DEN-BERG, J.W., KORT, W.J., TERPSTRA, O.T. & WILSON, J.H. (1992). Selective accumulation of endogenously produced porphyrins in a liver metastasis model in rats. *Gastroenterology*, **103**, 647-651.

VAN LEENGOED, H.L.L.M., VAN DER VEEN, N., VERSTEEG, A.A.C., OUELLET, R., VAN LIER, L.E. & STAR, W.M. (1993). In vivo photodynamic effects of phthalocyanines in a skin-fold observation chamber: role of central metal ion and degree of sulphonation. *Photochem. Photobiol.*, **58**, 575-580.

WEAGLE, G., PATERSON, P.E., KENNEDY, J. & POTTIER, R. (1988). The nature of the chromophore responsible for naturally occurring fluorescence in mouse skin. *J. Photochem. Photobiol., B*, **2**, 313-320.